# Implementation of Biopolymeric Nanomaterials to Reduce the Negative Impacts of Salinity on Tomato Quantity and Quality

**DOI:** 10.3390/molecules28041594

**Published:** 2023-02-07

**Authors:** Shreen S. Ahmed, Thana K. Khan, Gehan H. Abd El-Aziz, Tahsin Shoala, Hoda A. S. El-Garhy, Ashraf H. Fahmy

**Affiliations:** 1 Soil, Water and Environment Research Institute, ARC, Giza 12619, Egypt; 2 Department of Biological Sciences, Faculty of Science, King Abdulaziz University (KAU), Jeddah 21589, Saudi Arabia; 3 Environmental Biotechnology Department, College of Biotechnology, Misr University for Science and Technology, Giza 12563, Egypt; 4 Genetics and Genetic Engineering Department, Faculty of Agriculture, Benha University, Qalyubia 13736, Egypt; 5 Agricultural Genetic Engineering Research Institute, ARC, Giza 12619, Egypt

**Keywords:** tomato, biopolymeric nanomaterials, salinity, rice straw, starch, pectin

## Abstract

Sustainable waste reduction strategies and innovative waste reduction concepts, as well as their application in the creation of compounds and products with added value, can benefit the economy while reducing environmental pressures. This research aimed to use biopolymeric nanomaterials to reduce the negative effects of salinity on tomato yield and quality. Three types of biopolymers (cellulose, pectin, and starch) were synthesized and characterized using natural materials such as rice straw, orange peel, and potato peel. The polymer’s ability to retain sodium ions was investigated. A greenhouse experiment was conducted to assess the potential of natural polymers (cellulose, starch, and pectin individually or in combination) to reduce the salinity side effects on tomato plants (*Solanum Lycopersicon* L.) cultivar (Super Strain B). Tomato seeds were germinated on soil bits for 20 days before planting five seedlings in each pot (20 cm diameter) with three replicates and filling each pot with sandy loam soil, with or without natural polymers at a rate of 2 g/Kg. The results revealed that all the polymers utilized had a superlative capability to hold sodium ions for both soluble and exchanged sodium. The use of various natural polymer hydrogels increased the number and fresh weight of tomato fruits. Data showed that using biopolymers hydrogels reduced salinity stress by rising the content of phenol, flavonoid, and antioxidant enzymes such as catalase and peroxidase. The use of natural biopolymers significantly improved total soluble solids, pH, and juice substance. Implementing biopolymeric materials could reduce environmental pressures while increasing farm income. Innovative waste reduction strategies, such as the creation of value-added products, will benefit the economy, and this work is a good start in that direction.

## 1. Introduction

Tomato (*Solanum Lycopersicum)* represents one of the most significant vegetable plants on the planet. The global output exceeds 180 million metric tons [1]. Tomatoes have moderate salinity tolerance (1.3–6 dS m^−1^) [2], higher levels of EC diminish output and fruit size [3,4], as well as water and nutrient uptake [5]. Salinity stress causes reductions in root cell growth [6], leaf expansion [7], leaf chlorophyll [8], and plant photosynthesis, which has a negative impact on tomato plant growth [9].

Finding long-term useful solutions and potential agricultural waste applications is a current and highly coveted research goal. Sustainable waste reduction strategies and innovative waste reduction concepts, as well as their application in the creation of compounds and products with added value, can benefit the economy while reducing environmental pressures. Some issues must be resolved in the agriculture sector today. Waste has been used to make organic fuels, chemicals, animal feed, and other various products [10]. In the globally competitive environment, much emphasis is placed on investigating the efficacy of such waste.

Every year, approximately 250 million tons of inedible plant residues from crops (primarily cereals) are produced [11]. As a result, they have the potency to turn into a source of agricultural waste. This waste contains many fascinating materials (lipids, carbohydrates, and aromatic molecules) that can be used to make polymeric materials. When properly modified (as renewable sources), waste and manufacturing residues can yield intriguing new biopolymers [12]. Biopolymers are polymers manufactured or deduced from living organisms such as plants and microbes rather than petroleum, which is the traditional source of polymers.

While attention has recently been focused on the utilization of synthetic polymers, the worldview is already changing forward into natural polymeric nanomaterials. Biopolymer primary sources are renewable, which implies that they can decompose into carbon dioxide, methane, water, inorganic compounds, or biomass through the enzymatic action of microorganisms. Starch [13], chitosan [14], gelatin [15], lignin [16], cellulose, and k-carrageenan are several instances of biopolymers evaluated for application in sustained release [17].

Chitosan and cellulose are insoluble in water, whilst starch can be chemically modified to bring extra characteristics, making it ideal for agricultural applications. Natural polymers (cellulose, starch, and cellulose/starch) decreased the quantity of water irrigation required for tomato planting [18]. Natural polymeric nanoparticles can be employed in a variety of applications, such as nano herbicides, nano detectors, and nano fertilizers, to address constraints in the agricultural sector. These nano-scaled designed materials can assist in enhancing food production and nutritional content implicitly. Because of the major benefits and implementations of nano-systems, nanotechnology can revolutionize the agricultural sector while assisting in resolving major issues including food insecurity, crop production, and sustainable development [19].

Due to extreme climatic factors, the development of soil salinization and groundwater in recent decades has been a serious obstacle for agriculture in Egypt, adversely affecting agricultural production and therefore environmental sustainability. As a result, emerging innovations are pressingly necessary to accomplish greater sustainability and to guarantee the availability of safe food for the world’s population, which is expected to reach 9 billion by 2050. [20]. Hydrogel polymers can be utilized as soil amendments and carriers for slow-release fertilizers and pesticides. Hydrogels can be implemented by mixing with soil or sprinkling on the soil surface [21].

Biopolymers are polymers derived from plants. They are classified into three types: polynucleotides (DNA, RNA), polypeptides (proteins), and polysaccharides (e.g., carbohydrates). The low environmental impact, sustainability, non-toxicity, and low cost of biological stabilization methodologies inspired us to use biopolymers in geotechnical engineering [22]. Several studies showed that they have significant potential in construction and geotechnical soil-based applications [23,24]. Polysaccharide additives provide better quality control (reproducibility) and chemical versatility. Control over production, preparation, and addition methodologies is provided by their ex situ production, which can be accomplished through exo-cultivation or chemical extraction [25]. They are also the most abundant polymers on the planet, having been synthesized to perform a variety of biological functions (energy storage, structural support, gelling agents), providing a plethora of versatile chemical functionalities [26]. Polysaccharide additives have also been shown to be highly stable when exposed to saline conditions, across a wide pH range, and when stored for extended periods of time [27].

The application of hydrophilic polymers (0, 0.2, 0.6% *w*/*w*) to soils steadily increases soil moisture retention capabilities, yield, and plant-efficient use of water. Conversely, it reduces the negative effects of soil salinity on plants, and aids irrigation projects in arid and semi-arid areas [28]. Dehkordi (2017) investigated the effect of a hydrophilic polymer on salt and drought resistance in *Eucalyptus saligna* rooted cuttings and discovered that the polymer kept further water in the soil throughout water-stress conditions, encouraged salinity tolerance, and decreased drought stresses [29]. Furthermore, because of its high water-holding capacity, the polymer sustained Cl^-^ and Na^+^ in the soil solution. Due to the polymer’s salt- and water-holding capabilities, the polymer promoted greater resistance to salinity and drought stresses. As an innovative waste reduction strategy, this study was conducted to assess the potential of natural polymers nanomaterials (cellulose, starch, and pectin individually or in combination) created from farm wastes (rice straw, orange peel, and potato peel) to reduce the detrimental impact of salinity on the yield and quality of tomato plants (*Solanum Lycopersicon* L.) cultivar (Super Strain B) through in vivo and in vitro investigations.

## 2. Results and Discussion

Salinity is regarded as a significant factor that has a negative impact on plant growth. The goal of this study was to investigate the effect of biopolymers hydrogel application on mitigating the negative effects of salinity on tomato plants.

### 2.1. Biopolymers Characteristic

**Figure 1:**
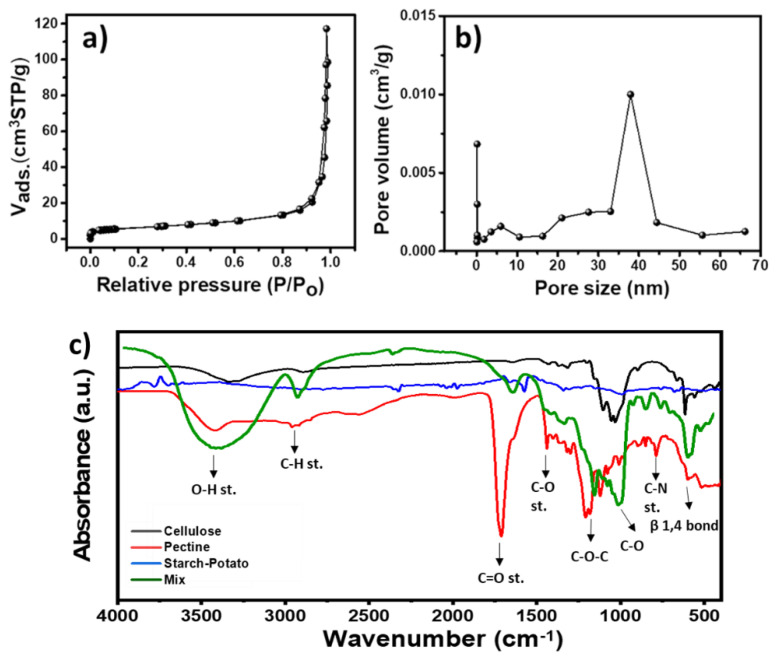
Surface analysis and pore size distribution of the polymer mixture (**a**,**b**). The FT-IR spectra of the cellulose, pectin starch-potato and a mix of them (**c**).

Figure 1a–c show surface analysis, pore size distribution, and FT IR characteristics of biopolymers nanomaterial.

#### 2.1.1. Surface Analysis and Pore Size Distribution

The nitrogen adsorption/desorption isotherms, as shown in Figure 1a, were used to investigate the surface analysis and pore size distribution. The isotherm of biopolymeric nanomaterials has a typical IV type, indicating a mesoporous surface. Furthermore, the isotherm has an H_3_ hysteresis loop caused by plate-like mesopores and slit-shaped pores. The surface parameter of a biopolymeric nanomaterial was estimated using the Brunauer–Emmett–Teller (BET) model after analyzing adsorption data. Biopolymeric nanomaterial has a specific surface area of 32.15 m^2^/g.

The total pore volume was determined to be 0.24 cm^3^/g. The pore size distribution was estimated from the desorption data analysis using the BJH model via the nonlocal density functional theory (NLDFT) method, as shown in Figure 1b. The analysis reveals the presence of mesopores with an average pore size distribution of 39.4, which is advantageous for ion movement.

#### 2.1.2. The FT-IR Spectra

Figure 1c depicts the FT-IR spectra. The absorption peak in the region of 1060–1250 cm^−1^ in the cellulose spectrum is a significant feature of C-O and C-O-C. The peak at 615 cm^−1^ represents the β1,4 bond’s typical breathing mode. Furthermore, the pectin spectrum shows a peak at 3421 cm^−1^ for O-H stretching and 2918, 1690, 1299, 1207, 1122, 764 cm^−1^ for C-H stretching of alkanes, C=O stretching of alkynes, C-O stretching of esters or carboxylic acid, C-N stretching, and C-Cl stretching, respectively. In addition, the starch–potato spectrum shows a peak at 992 cm^−1^ for the characteristic starch backbone, as well as peaks at 1574, 1659, and 2325 cm^−1^ for O-H bending, C-C aromatic ring stretching, and C-H stretching, respectively. Furthermore, the spectrum of the mixing of all biopolymers reveals the same identification peaks of cellulose, starch, and potato, confirming the successful mixing of all materials.

### 2.2. Incubation Experiment

This experiment was carried out to determine the efficiency of polymers in mitigating salt stress. After 30 days of incubation, the EC of the soil treated with various doses of salt and polymers hydrogel application was measured. The highest increasing rate in EC was measured at the highest doses (5 dS/m) in non-treated soil with polymers hydrogel (pectin, cellulose, starch, and a mixture of all of them) (Table 1). The results showed that the polymers (pectin, cellulose, starch, and a mixture of them) had a positive effect on salinity levels.

At 3 dS/m, the percentage of decrease of soil salinity reached 3.14, 4.86, 6.00, and 10.00%, respectively, compared to control (tap water); while, at 5 dS/m, the percentage of decrease of soil salinity reached 1.48, 2.96, 3.89, and 5.93%, respectively. Aydin et al. (2011) found that adding hydrogel to saline soil improved the variables affected by high salinity and reduced soil electrical conductivity [30].

### 2.3. Evaluation of the Polymer’s Efficiency in Retaining Sodium Ions (ppm)

The data in Table 2 revealed the polymer’s efficiency in retaining sodium ions (parts per million). After 24 h, the Na ions of polymers hydrogel treated with 50 mL of salt solution (3 dS/m) were measured. All the polymers used had a significant effect to retain sodium ions for both soluble sodium and exchanged sodium, according to the results. The ability to retain sodium ions varied according to polymer type and weight, with 1 gm of each polymer retaining the highest percentage of sodium ion retention compared to 0.5 gm of each polymer. For Soluble-Na, the percentage of sodium ions retained by different polymers (pectin, cellulose, starch, and a mixture of all of them) at 0.5 gm was 37, 39.4, 42.4, and 53.6%, respectively, while at 1.0 gm, the percentage of sodium ion retention was 50.6, 53.5, 64.5, and 81.8%. Regarding Exchange-Na, the percentage of sodium ions retained by different polymers (pectin, cellulose, starch, and a mixture of all of them) at 0.5 gm was 17.4, 19.1, 21.4, and 24.8%, respectively, while at 1.0 gm, the percentage of sodium ion retention was 24.6, 27.6, 31.2, and 34.7%. From the results, it is seen that Na^+^ can be absorbed by the polymer and migrate into the gel matrix. These findings are consistent with those of Sánchez et al. (2020), who provided an overview of versatile biopolymer materials with various functional groups for the removal of hazardous inorganic species [31]. These biological sorbents are completely water-soluble and versatile, allowing them to achieve higher sorption capacity and efficacy under a variety of experimental conditions when used in conjunction with ultrafiltration membranes via liquid phase retention polymer (LPR) technology. These heavy-duty sorbents are generally more environmentally friendly and biodegradable than more commonly used synthetic polymers.

### 2.4. The Effect of Biopolymers Hydrogel on the Number and Fresh Weight of Fruits

The number and fresh weight of tomato fruits were reduced due to salinity stress. The application of various natural polymers hydrogel (pectin, cellulose, starch, and a combination of all of them) increased both the number and the fresh weight of fruits.

**Figure 2:**
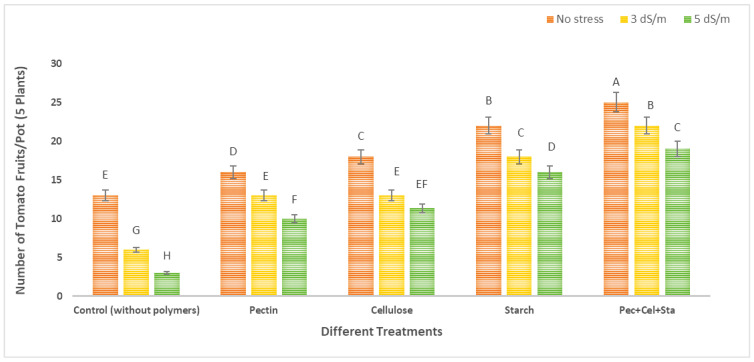
Effect of natural polymers hydrogel on the number of tomato fruits/pot (5 plants) under salinity stress. The means of each column with different letters are significantly different at *p* ≤ 0.05. Results are shown as mean ± standard error obtained from three replicates.

**Figure 3:**
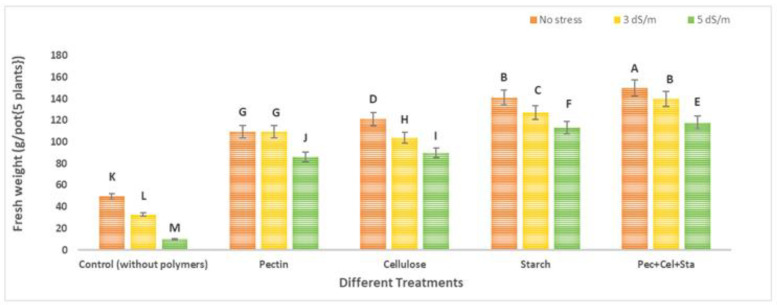
Effect of natural polymer hydrogel on fresh weight (g/pot {5 plants}) of tomato under salinity stress. The means of each column with different letters are significantly different at *p* ≤ 0.05. Results are shown as mean ± standard error obtained from three replicates.

Figure 2 and Figure 3 show the results of tomato growth (number of fruits and fresh weight). The means of each column with different letters are significantly different at *p* ≤ 0.05. The number of fruits and fresh weight were significantly decreased in plants grown in soil under salinity stress conditions untreated with natural polymers. The greatest decrease was observed at high salinity (5 dS/m). When compared to the control, the maximum decrease in the number of fruits and fresh weight was 76.9 and 80.1%, respectively.

Data showed that using different natural polymers hydrogel under investigation (pectin, cellulose, starch, mixture of all of them) in soil with different levels of irrigation water salinity (tap water as no stress 0.2 dS/m, 3 dS/m, and 5 dS/m) increased the number of fruits and fresh weight significantly. The number of fruits was increased by using different polymers hydrogel (pectin, cellulose, starch, and a mixture of all of them) relative to the control (untreated with polymers hydrogel); these increases were 2.2, 2.2, 3.0, and 3.7-fold respectively for salinity stress 3 dS/m, and 3.3, 3.8, 5.3, and 6.3-fold respectively for salinity stress 5 dS/m. For the fresh weight of fruits, there was an increase due to the use of different polymers hydrogel (pectin, cellulose, starch, and a mixture of all of them) compared to the control (untreated with polymers hydrogel); these increases were 3.3, 3.2, 3.9, and 4.3-fold, respectively, that were controlled under salinity stress 3 dS/m, and 8.7, 9.1, 11.5, and 12.0-fold, respectively, that were controlled under salinity stress 5 dS/m. Plants have generally adapted to osmotic and ionic stress under salt stress. Hyperosmotic shock and ionic imbalance are two of the negative effects of high salt concentrations on plant cells [32]. These findings are consistent with those of Shokuohifar et al. (2016), who found that using SAP reduced the negative effects of saline irrigation (4.0 and 6.0 dc m^−1^) in sandy-clay soils by increasing porosity and water available to the plant by up to 40.8% and two times, respectively, over the control [33].

Furthermore, salinity alters the physiological and biochemical processes of plants. These changes appear in plants depending on the effects of ions and solutes in the root zone on water activity in the cell and physiological and biochemical functions of the cell, reducing turgor, limiting photosynthesis, and increasing ion deficiency due to an inadequate transport mechanism [34]. Plants grown in substrates amended with hydrophilic polymers wilted more slowly than plants grown in an unaltered medium [35].

Other evidence suggests that the presence of polymers increases plant survival, water use efficiency, and dry matter production during drought and salinity conditions. It is worth noting that the biopolymer may activate the microorganisms in the soil, which helps to facilitate the nutrients in the soil in the root zone, allowing the plant to absorb them, which affects the efficiency of the metabolism process. The effects of hydrogel application on crops grown in substrates have recently been studied under saline conditions. Hydrogel amendment improved wheat emergence [36], maize pollen germination [37], and salt tolerance and growth of horticultural crops such as barley, tomato, cucumber, and lettuce.

Hydrogel polymers promote plant growth by increasing soil water holding capacity and extending the time until wilting occurs, increasing plant survival under water stress [38], decreasing fruit drop ratio, and potentially increasing total yield and fruit weight under various severity conditions [39]. Furthermore, adding hydrogel to the soil increased plant circumference; this could be attributed to an increase in the amount of available water in the root zone, implying longer irrigation intervals [40].

Furthermore, the application of hydrogel polymer is used to create a water reservoir near the root zone of plants, decrease the osmotic moisture of soil, improve the capacity of plant-available water, enhance plant growth, increase the overall yield, and reduce crop production costs. Hydrogels are used to improve plant viability, seed germination, ventilation, and root development, primarily in arid environments [41]. Furthermore, in terms of plant growth, it has been observed that the use of hydrogel results in a significant increase in plant growth [42].

### 2.5. The Effect of Biopolymers Hydrogel on Phenol and Flavonoid Content of Tomato Fruits under Salinity Stress

**Figure 4:**
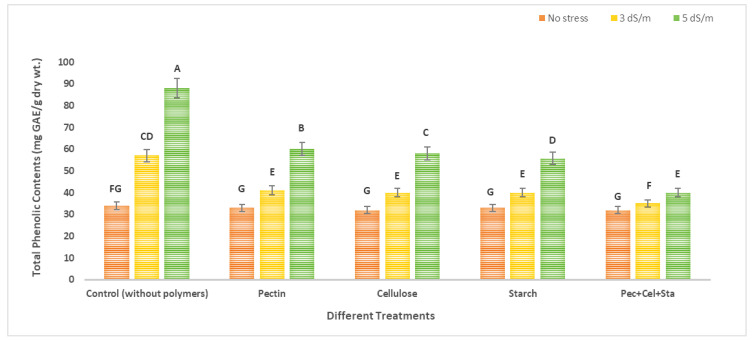
Effect of biopolymers hydrogel on total phenolic contents (mg GAE/g dry wt.) content of tomato fruits under salinity stress. The means of each column with different letters are significantly different at *p* ≤ 0.05. Results are shown as mean ± standard error obtained from three replicates.

**Figure 5:**
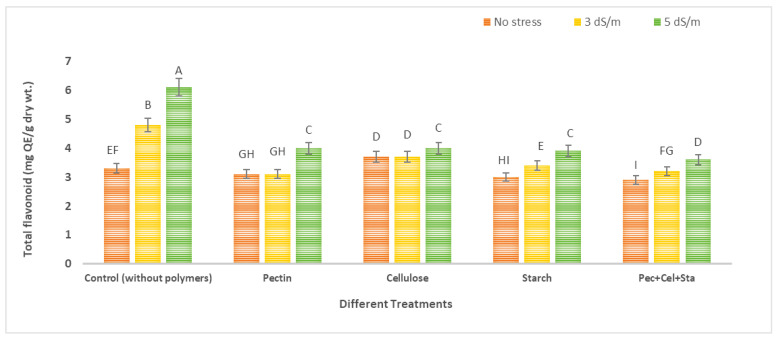
Effect of biopolymers hydrogel on flavonoid (mg QE, quercetin equivalent)/g dry wt.) content of tomato fruits under salinity stress. The means of each column with different letters are significantly different at *p* ≤ 0.05. Results are shown as mean ± standard error obtained from three replicates.

Figure 4 and Figure 5 show data of tomatoes’ phenol and flavonoid content. Statistical analysis revealed that salinity levels and biopolymers hydrogel application had a significant effect on phenol and flavonoid levels. The means of each column with different letters are significantly different at *p* ≤ 0.05. The highest levels of phenol and flavonoid were found in plants exposed to salinity stress in untreated soil by biopolymers. The greatest increase was observed at high salinity (5 dS/m). When phenol and flavonoid levels were compared to controls, the maximum increase was 2.6 and 1.8-fold, respectively. In contrast, the application of different biopolymers hydrogel (pectin, cellulose, starch, or a mixture of all of them) in soil with different levels of irrigation water salinity (tap water as no stress 0.2 dS/m, 3 dS/m, and 5 dS/m) decreased phenol and flavonoid content.

For phenol, phenol content was reduced by using different polymers hydrogel (pectin, cellulose, starch, and a combination of all of them) relative to the control (untreated with biopolymers under stress). These reductions reached 28.1, 29.8, 29.8, and 38.6%, respectively, compared to the control under salinity stress 3 dS/m, and 53.4, 54.5, 54.5, and 60.2%, respectively, compared to the control under salinity. Using the same approach, for flavonoids, there was a decrease when using different polymers hydrogel (pectin, cellulose, starch and mixture all of them) relative to control (untreated with polymers hydrogel under stress); these decreases reached 35.4, 22.9, 29.2 and 33.3%, respectively, that were controlled under salinity stress 3 dS/m, while being 34.4, 34.43, 36.1 and 40.9%, respectively, compared to control under salinity stress 5 dS/m.

Many researchers concur that increases in phenol and flavonoid concentrations occur under salt-stress circumstances. Total phenol and flavonoid are a group of secondary metabolites that perform cellular signaling functions under abiotic stress conditions, and may operate as antioxidants to protect the plant from oxidative stress [43,44]. Hydrogel reduces the effect of salt stress on plant growth, and decreases phenol and flavonoid concentration [18].

### 2.6. The Effect of Biopolymers Hydrogel on Tomato Catalase and Peroxidase Activity under Salt Stress

**Figure 6:**
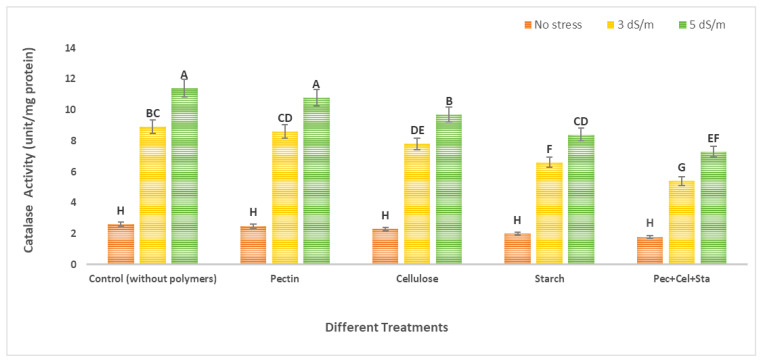
Effect of natural polymers hydrogel on catalase enzyme of tomato fruits under salinity stress. The means of each column with different letters are significantly different at *p* ≤ 0.05. Results are shown as mean ± standard error obtained from three replicates.

**Figure 7:**
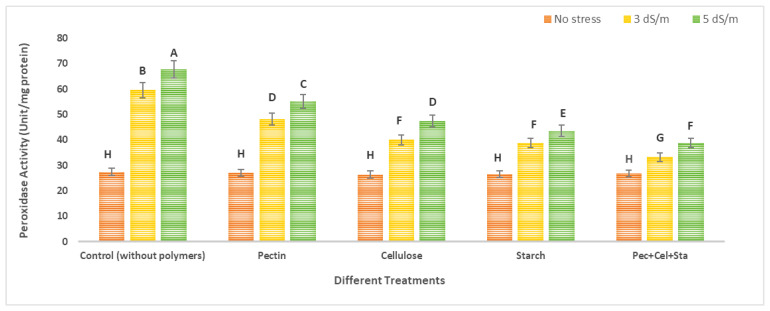
Effect of natural polymers hydrogel on peroxidase activity of tomato fruits under salinity stress. The means of each column with different letters are significantly different at *p* ≤ 0.05. Results are shown as mean ± standard error from three replicates.

Figure 6 and Figure 7 depict information on the catalase and peroxidase activity in tomato, respectively. Data showed that using biopolymers hydrogels had a favorable impact on lowering salinity stress by enhancing antioxidant enzymes, which are crucial in defending the plant cell against salinity stress-related damage. The plants exposed to salt stress conditions in untreated soil by biopolymers showed the highest catalase and peroxidase activity. At a high salinity (5 dS/m), the greatest rise was seen. For catalase and peroxidase, the maximal increases were 4.3 and 2.4 times greater than the control, respectively. In contrast, the application of various biopolymers hydrogel (pectin, cellulose, starch, or a combination of all of them) in soil with varying salinities of irrigation water (tap water as no stress, 0.2 dS/m, 3 dS/m, and 5 dS/m) revealed a decrease in the content of catalase and peroxidase activity.

For catalase activity, different hydrogel polymers (pectin, cellulose, starch, and mixtures of all of them) were used to reduce catalase activity in comparison to the control (which was not treated with biopolymers under stress). These decreased percentages reached 3.4, 12.4, 25.8, and 39.3%, respectively, compared to the control under salinity stress 3 dS/m and were 5.3, 14.9, 26.3, and 35.9%, respectively, compared to peroxidase activity, and decreased when pectin, cellulose, starch, or a combination of them were used in comparison to the control (not treated with polymers hydrogel under stress). These decreases were 19.1, 32.9, 34.9, and 44.3%, respectively, for the control under salinity stress 3 dS/m, and 18.5, 29.9, 35.6, and 42.7%, respectively, for the control under salinity stress 5 dS/m.

The production of reactive oxygen species (ROS), which causes oxidative damage to macromolecules such as lipids and proteins and ultimately results in cell death, has generally been shown in various investigations. Plants produce antioxidants in response to stresses such as salt [45]. Plant cells produce antioxidant enzymes such as peroxidase and catalase to defend against oxidative damage [46]. By enhancing the water holding capacity of modified media, which have been utilized to support plant establishment and growth, hydrogels can reduce salt stress. The existence of polymers extends plant longevity, improves water usage efficiency, and enhances dry matter synthesis amid drought and salinity conditions, according to additional research.

Under saline circumstances, the effects of hydrogel treatment on crops grown in substrates have been studied. Certain horticultural crops, such as barley, tomato, cucumber, and lettuce, benefited from hydrogel amendments that increased their salt tolerance and growth. According to Aydin et al. (2011) [30], using hydrogel under salinity stress caused a decrease in enzyme activity as the salinity stress increased. Although a substantial relationship was discovered, it is important to note that treatment with hydrogel application tended to lessen the impact of salt stress on the enzymes’ activity [30].

When compared to the control, bean plants cultivated in high salinity conditions showed enhanced enzyme activity in the leaves. CaCl_2_ and NaCl had the greatest enzyme activity values, respectively. However, the salt effect was lessened, and this enzyme activity was reduced by the hydrogel application therapy [30]. By enhancing the soil’s characteristics or indirectly by increasing plant metabolism to withstand excess salts, hydrogel compounds can reduce salt stress in crop plants. Sometimes, direct and indirect impacts may contribute to a plant’s ability to adapt to saline environments [47].

### 2.7. Impact of Biopolymers Hydrogel on the Quality of Tomato Fruits under Salt Stress

Table 3 shows how salt stress and natural biopolymers affect tomato fruit quality. With the application of natural biopolymers, total soluble solids, pH, and juice content were considerably improved. Utilizing different biopolymers hydrogel (pectin, cellulose, starch, and a combination of all of them) enhanced total soluble solids, pH, and juice levels compared to controls (untreated with biopolymers under stress). Similar to this, Sender Kumaran (2016) also noted that the use of hydrophilic polymer increased the ascorbic acid content, TSS, pH, lycopene content, and juice content of tomatoes. Increased metabolic activities result in the synthesis of large amounts of acids, metabolites, and glucose, which may be the cause of TSS increases by the polymer. [48].

These reserves, which were created, could have influenced the initial makeup of TSS [49]. Additionally, they found that the use of hydrophilic polymer raised the pH of tomato fruits. When fruits are subjected to water stress, the pH of the fruit may decrease because of changes in the malic and citric acid content [50,51]. According to Shreen and Fahmy (2019), applying natural polymers significantly improved vitamin C, total soluble solids, pH, lycopene, and juice content [18].

## 3. Materials and Methods

### 3.1. Natural Polymer Preparation

Three types of biopolymers were synthesized and characterized using natural ingredients such as rice straw, orange peel, and potato peel. Cellulose and starch substances under investigation have previously been described in our previous work [18].

### 3.2. Starch Polymer Preparation

Potato peels were cleaned, cut, and dried before being dispersed in 1% sodium Meta bi sulphate in 100 mL of distilled water, as Vasanthan (2001) described. A muslin cloth was used to filter the suspension. The suspension has been centrifuged for 20 min at 5000 rpm. The starch that had been allowed to settle at the bottom of the centrifuge tube was drained with toluene, oven-dried at 30° to 40 °C, and milled into a fine powder with a mortar and pestle [52].

### 3.3. Preparation of Cellulose Polymer Hydrogel

For cellulose preparation, 50 g of rice straw was mixed with 5% NaOH and ultrasonically cooked at 170 °C for 2 h. The fiber was washed with distilled water before being added to 100 mL of distilled water in a flask of 250 mL with a magnetic stirrer, and placed in a 95 °C water bath for 30 min. At 60 °C, potassium persulfate was added to the treated cellulose. The temperature was then raised to 70–80 °C till the white color gel was achieved. The white gel was washed with methanol for 30 min, followed by ethanol for 5 min, and dried in an oven at 60 °C until the fixed weight was obtained.

### 3.4. Pectin Hydrogel Preparation

Ten grams of fresh orange peel were added to the citric acid solution (distilled water was mixed with citric acid until the pH reached 2), and the solution was heated at 80 °C for 10 min. Following cooling, the solution was filtered. The pectin precipitate was treated with ethanol. The solution was filtered through a fine filter cloth to separate the jelly pectin, which was then dried at 50 °C for two hours. After preparing all the polymers in the laboratory, equal weights of each polymer were weighed separately, and mechanically mixed, and then surface analysis and pore size distribution were studied (Pandharipande et al., 2012) [53].

### 3.5. Incubation and Measuring of Soil Electrical Conductivity (EC) Experiment

One kilogram of sandy loam soil has been placed in each pot. Each natural polymer hydrogel (pectin, cellulose, starch, and a combination of all of them) was added in 0.6 g increments and thoroughly mixed. In addition to tap water (0.2 dS/m) as a control, all pots were watered with seawater diluted to 3 and 5 dS/m levels until the soil was saturated. Soil samples from each pot were collected after 30 days of salt and hydrogel application, and electrical conductivity (EC) was measured.

### 3.6. Laboratory Experiment to Determine the Polymer’s Ability to Retain Sodium Ions

In 50 mL of saline solution (3 ds/m), 0.5 or 1.0 g of each hydrogel polymer was placed. The polymer solution was shaken and left overnight. Each polymer was given its filter. According to Page et al. (1982), soluble and Na ion exchange were estimated using a flame photometer [54]. 

### 3.7. Greenhouse Experiment 

During two successive seasons (2020/2021), a greenhouse experiment was conducted at the Greenhouse and Experimental Farm, Agricultural Genetic Engineering Research Institute, Agricultural Research Center, Giza, Egypt, to assess the potential of natural polymers (cellulose, starch, and pectin individually or in combination) to reduce the side effect of salinity on tomato. Tomato seeds (*Solanum Lycopersicon L*.) cultivar (Super Strain B) were germinated on soil bits for 20 days before five seedlings were planted in each pot (20 cm diameter) with three replicates, and each pot was filled with sandy loam soil, with or without natural polymers, at a rate of 2 g/Kg [55]. Until the trial ended (6 months), all plant pots were watered with seawater diluted to levels of 3 dS/m and 5 dS/m, in addition to tap water (0.2 dS/m) as a control (no stress). The trial was a split plot with randomized complete blocks. Table 4 shows the physical and chemical properties of the soil studied before planting.

### 3.8. Analytical Methods

The soil was analyzed following Page et al. (1982), and the total soluble solids in fresh fruit were estimated using an Erma hand refractometer for plant analysis [54]. The juice content was calculated and expressed based on the total weight of the fruits. A digital pH meter was used to determine the pH of tomato fruit juice. Total flavonoids and phenol were determined by colorimetric methods according to Wei and Intan [56], and Singleton and Rossi [57].

Catalase activity was determined by measuring the disappearance of H_2_O_2_ at 240 nm (ε = 40 mM^−1^ cm^−1^) using the Aebi method (1984) [58]. Then, 50 mM K-phosphate buffer (pH 7.0), 33 mM H_2_O_2_, and enzyme extract were added to the reaction mixture. Peroxidase activity was measured at 436 nm by its ability to convert guaiacol to tetraguacol (ε = 26.6 mM^−1^ cm^−1^) using the methodology of Polle et al. (1994) [59]. Next, 100 mM K-phosphate buffer (pH 7.0), 20.1 mM guaiacol, 10 mM H_2_O_2_, and enzyme extract were added to the reaction mixture. The addition of H_2_O_2_ at 436 nm for 5 min increased absorbance. All measurements were made in triplicate, and data were represented as mean values with standard deviations on a dry weight basis for all analyses except for the enzyme, which was estimated in fresh fruits. All plants were collected at the end of the season, where the number and weight of fruits per pot were recorded.

Protein concentrations were determined utilizing Bradford’s (1976) [60] method with BSA as a standard.

### 3.9. Statistical Analysis

The statistical analysis was carried out using two-way ANOVA using SPSS, ver. 25 (IBM Corp. Released 2013). Data were treated as split plot with randomized complete blocks according to Steel et al. (1997) [61]. Experiments were conducted in three replicates. Duncan’s new multiple-range test was used to differentiate the obtained means [62]. The significance level was set at <0.05.

## 4. Conclusions

Sustainable waste reduction approaches and innovative waste reduction notions, as well as their use in the development of substances and products with additional value, can enhance the economy while minimizing environmental stressors. Biopolymeric materials could be used to reduce environmental stressors while boosting agricultural production. Innovative waste reduction strategies, such as the development of value-added products, will benefit the economy, and this work is a good starting point. Furthermore, making the best use of plant leftovers aids in the production of beneficial substances such as biopolymers, which are made from rice straws, potato skins, and orange peels. Because of extreme weather conditions that have a negative impact on agricultural production and, as a result, sustainable growth, soil salinization has become a significant issue for agriculture. The biopolymer hydrogel (cellulose, pectin, starch, and a combination of all three) reduced the growth-impairing effects of salt on tomatoes.

## Figures and Tables

**Table 1:** Polymer efficiency to mitigate salt stress by evaluating soil electrical conductivity (EC), (dS/m).

Polymer	Level of Salinity (dS/m)			Mean of Polymer
	0	3	5	
Soil without polymer	0.36 ± 0.01 ^aC^	3.50 ± 0.12 ^aB^	5.40 ± 0.00 ^aA^	3.09 ± 0.74 ^a^
Soil with cellulose	0.34 ± 0.01 ^aC^	3.39 ± 0.01 ^aB^	5.32 ± 0.68 ^aA^	3.02 ± 0.67 ^a^
Soil with pectin	0.33 ± 0.01 ^aC^	3.33 ± 0.01 ^abB^	5.24 ± 0.01 ^abA^	2.97 ± 0.82 ^a^
Soil with starch	0.32 ± 0.01 ^aC^	3.29 ± 0.01 ^abB^	5.19 ± 0.02 ^abA^	2.93 ± 0.71 ^a^
Soil with mixture polymer	0.30 ± 0.01 ^aC^	3.15 ± 0.02 ^bB^	5.08 ± 0.02 ^bA^	2.84 ± 0.69 ^a^
Mean of level	0.33 ± 0.01 ^C^	2.73 ± 0.32 ^B^	5.11 ± 0.13 ^A^	
	Correlation			
	**Value**			
	**Corr.**		**Sig.**	
Polymer	−0.025		0.872	
Level	0.972 ***		0.000	

a and b: There is no significant difference (*p* > 0.05) between any two means, within the same column and which have the same superscript letter; A, B & C: There is no significant difference (*p* > 0.05) between any two means, within the same row, and which have the same superscript letter. *** Correlation is significant at a 0.000 level.

**Table 2:** Evaluation of the polymer’s efficiency in retaining sodium ions (ppm).

	* Salt (3 dS/m)							
Polymer	Soluble-Na				Exchange-Na			
	0.5	1.0		Mean	0.5	1.0		Mean
Cellulose	26.41 ± 0.24 ^cB^	35.82 ± 0.26 ^cA^		31.12 ± 2.12 ^c^	12.82 ± 0.10 ^cB^	18.45 ± 0.29 ^cA^		15.64 ± 1.27 ^d^
Pectin	25.14 ± 0.11 ^dB^	33.91 ± 0.25 ^dA^		29.53 ± 1.97 ^d^	11.63 ± 0.06 ^dB^	16.44 ± 0.13 ^dA^		14.04 ± 1.08 ^d^
Starch	28.36 ± 0.11 ^bB^	43.20 ± 0.17 ^bA^		35.78 ± 3.32 ^b^	14.30 ± 0.10 ^bB^	20.88 ± 0.11 ^bA^		17.59 ± 1.47 ^b^
Mixture	35.91 ± 0.09 ^aB^	54.80 ± 0.48 ^aA^		45.36 ± 4.23 ^a^	16.62 ± 0.07 ^aB^	23.21 ± 0.42 ^aA^		19.92 ± 1.49 ^a^
Mean	28.96 ± 0.36 ^B^	41.93 ± 2.48 ^A^			13.84 ± 0.16 ^B^	19.75 ± 0.78 ^A^		
				Correlation				
	**Soluble-Na**				**Exchange-Na**			
	**Corr.**		**Sig.**		**Corr.**		**Sig.**	
Polymer	0.634 **		0.001		0.590 **		0.002	
Level	0.705 ***		0.000		0.795 ***		0.000	
Soluble-Na					0.969 ***		0.000	

a, b, c and d: There is no significant difference (*p* > 0.05) between any two means, within the same column, and which have the same superscript letter; A and B: There is no significant difference (*p* > 0.05) between any two means, within the same row, and which have the same superscript letter. * Salt (3 dS/m) equals 66.96 ppm Na. ** Correlation is significant at a 0.001 level. *** Correlation is significant at a 0.000 level.

**Table 3:** Effect of biopolymers hydrogel on tomato fruits quality under salinity stress.

Treatments	Level of salinity	pH	TSS%	Juice%
Control (Without polymer)	0.0	4.010 ^g^	4.200 ^e^	54.62 ^d^
	3 dS/m	3.640 ^i^	3.190 ^k^	50.66 ^h^
	5 dS/m	3.060 ^j^	2.950 ^l^	36.73 ^j^
Pectin	0.0	4.490 ^c^	4.290 ^d^	55.44 ^c^
	3 dS/m	4.140 ^f^	3.640 ^i^	52.11 ^f^
	5 dS/m	3.820 ^h^	3.450 ^j^	48.93 ^i^
Cellulose	0.0	4.610 ^b^	4.590 ^c^	56.74 ^b^
	3 dS/m	4.220 ^e^	3.953 ^g^	52.36 ^f^
	5 dS/m	4.040 ^g^	3.670 ^i^	50.19 ^h^
Starch	0.0	4.650 ^b^	4.830 ^b^	57.46 ^a^
	3 dS/m	4.290 ^d^	4.180 ^e^	53.43 ^e^
	5 dS/m	4.110 ^f^	3.900 ^h^	51.23 ^g^
Mix of all polymers	0.0	4.740 ^a^	4.940 ^a^	56.53 ^b^
	3 dS/m	4.300 ^d^	4.310 ^d^	53.87 ^e^
	5 dS/m	4.200 ^e^	4.110 ^f^	52.07 ^f^

a, b, c, d, e, f, g, h, i, j, k and l: There is no significant difference (*p* > 0.05) between any two means for each attribute, within the same column, and which have the same superscript letter.

**Table 4:** Some characteristics of original soil experiment in El Giza Agricultural Research Center.

Characteristics	Value
Physical properties	
Particle size distribution	
Sand%	60
Silt%	25
Clay%	15
Texture soil	Sandy loam
Chemical properties	
Organic matter content%	1.24
pH (1: 2.5)	7.80
EC ds/m	1.61
Cations meq/L	
Ca^++^	3.80
Mg^++^	3.31
Na^+^	4.60
K^+^	0.26
Anions meq/L	
CO^=^_3_	0.00
HCO^−^_3_	1.60
CL	7.21
SO^=^_4_	3.17

## Data Availability

The data presented in this study are available upon request from the corresponding author. The data are not publicly available due to privacy concerns.
